# Reply to Vorland et al. Mixed Random and Nonrandom Allocation, and Group Randomization Have Been Mislabeled and Misanalysed, Necessitating Reanalysis. Comment on “Conner et al. KiwiC for Vitality: Results of a Randomized Placebo-Controlled Trial Testing the Effects of Kiwifruit or Vitamin C Tablets on Vitality in Adults with Low Vitamin C Levels. *Nutrients* 2020, *12*, 2898”

**DOI:** 10.3390/nu14194065

**Published:** 2022-09-30

**Authors:** Tamlin S. Conner, Benjamin D. Fletcher, Jillian J. Haszard, Juliet M. Pullar, Emma Spencer, Louise A. Mainvil, Margreet C. M. Vissers

**Affiliations:** 1 Department of Psychology, University of Otago, Dunedin 9054, New Zealand; 2 Biostatistics Centre and Division of Sciences, University of Otago, Dunedin 9054, New Zealand; 3 Centre for Free Radical Research, Department of Pathology and Biomedical Science, University of Otago, Christchurch 8140, New Zealand; 4 Department of Pathology and Biomedical Science, University of Otago, Christchurch 8140, New Zealand; 5 Department of Human Nutrition, University of Otago, Dunedin 9054, New Zealand

We have read the Comment article by Vorland et al. [1], based on our original KiwiC for Vitality trial article and are pleased to have the opportunity to respond. Vorland et al. have produced this Comment following an examination of our data and methodology. This was made possible by our sharing of the original data, code, and study details with them upon request. This is acknowledged by the authors, who noted our “collegiality in communication” and that we “expeditiously shared [our] data, code and additional detail on [our] design”. Our sharing of the data reflects our commitment to open science practices and our belief that science is strengthened through replication and good-faith interrogation. 

In their audit of our trial, the authors identified two potential issues with our original paper. The first was that we did not consider possible statistical dependencies arising from group randomisation, where we randomly assigned people attending a clinic on a given day to either kiwifruit or tablets. In our original paper, we detailed the rationale for using a group randomisation model. This was done to enable the allocation of the intervention condition to be delivered to individual participants without substantial risk of contamination, i.e., to avoid participants being exposed to the other conditions by seeing others receiving kiwifruit when they received tablets or vice versa. The potential for impacting on their mood and well-being, as two of our three primary outcome measures, was considered to be a substantial risk if the participants were to make a prior judgement as to the perceived superiority or inferiority of their allocation (fruit vs. tablets). Hence, the rationale for a group randomisation model was strong, and is in line with statistical methods recommendations, as indicated by Turner et al.: “a group randomised trial is the best comparative design available if the intervention…. cannot be delivered to individual members of the group without substantial risk of contamination across study arms…” [2]. 

There were several reasons we did not initially account for statistical dependency in our randomisation groupings. We did not view clinic days as meaningful units that could give rise to dependencies. As described above, the study participants were drawn from a single population, run at the same clinic site location (not different site locations), and participants in each clinic did not interact with each other or know each other. These were simply clinic slots run in the same on-campus location from Monday to Thursday, with some variation due to holidays, and appointment slots were allocated in a blinded manner. We were surprised that the audit group likened this design to the PREDIMED trial [3,4], which had 11 different data collection sites and enrolled couples with household-linked dependencies. Nevertheless, after discussing our design with an independent biostatistician following this audit, we agree that from a statistical perspective, our data were clustered. Reanalysis would test whether the randomisation process could have resulted in dependencies arising from people in any one clinic being more similar to each other than to those in another clinic (though through what mechanisms, we are not entirely sure).

The second issue identified by Vorland et al. was that we introduced some non-random allocation into our study. This non-random selection of participants stemmed from allocating the first two weeks of participants to the kiwifruit condition due to circumstances outside our control (late arrival of the tablets from overseas). This was a pragmatic decision reflecting the messiness of real-world constraints in a clinical trial. However, all participants were from a single population of regular tertiary students with regular class schedules. They were assigned into clinics by a clinic coordinator without regard to their individual characteristics and without knowing which clinic would be receiving which intervention. The study clinic co-ordinator and the participants were blinded to the allocation until they completed the second clinic appointment. Again, despite our pragmatic approach, we understand that this introduces non-random allocation to our design. 

In response to the Comment, and the concerns they raised, we engaged the services of an independent biostatistician (JJH) who reviewed our design and then completed an extensive reanalysis of the data, taking into account the dependencies in observations using mixed effects modelling and further strengthening our data presentation to adhere to CONSORT guidelines. This included removing a figure showing within-group comparisons and replacing it with a figure showing between-group comparisons. The result of this exercise is that the pattern of our results was essentially unchanged. The positive effects of the vitamin C and kiwifruit interventions on subjective vitality outcomes were mostly the same. There were minor changes in the effect sizes, but both vitamin C and kiwifruit were found to improve markers of vitality, whereas there were no effects in the placebo tablet group. The overall conclusions of the study are unaltered. 

The authors of the Comment suggested that we retract the original paper and republish it with reanalysed data. We did attempt this, but the Editors of Nutrients considered that the reanalysis did not warrant a ‘Retract and Replace’, as the conclusions of the paper were essentially unchanged. Hence, this circumstance does not meet the criteria for retraction. In response, we suggested to submit a reanalysis of the data as a separate paper. However, this request was declined on the grounds that a reanalysis paper would be too close to a duplication of the original publication, despite our pointing out that the new paper would focus only on the reanalysis and that there is precedent for publishing reanalysed data. The Editors finally agreed to a correction, which we have now submitted. 

We have undertaken this exercise to ensure clarity of the scientific record, to address the two issues raised by Vorland et al., and to demonstrate our confidence in the data. We note that the original paper was subject to extensive peer review, yet no matters of concern about the randomisation schedule or analyses were raised during the peer review process. In light of these changes, we suggest that researchers refer to the KiwiC for Vitality trial as a placebo-controlled trial rather than a randomised controlled trial and cite our corrected KiwiC for Vitality paper going forward [5].

## Data Availability

The data used for this Comment article are from the associated manuscript. Access to this data is available upon reasonable request, in collaboration with the authors of the study.
